# Identification of upregulated genes in pulmonary hypertension using RNA sequencing profiling

**DOI:** 10.6026/973206300213105

**Published:** 2025-09-30

**Authors:** Jino Blessy J., Jayasurya R., Shanmugapriya Murugan, Shirley Lois G.

**Affiliations:** 1 Department of Bioinformatics, Sri Ramachandra Faculty of Engineering and Technology, Sri Ramachandra Institute of Higher Education and Research, Chennai 600116, India

**Keywords:** Pulmonary hypertension, next-generation sequencing, RNA sequencing, differential gene expression, protein-protein interactions, hub genes

## Abstract

Pulmonary hypertension (PH) is a chronic and progressive disease that is characterized by increased pulmonary arterial blood pressure, causing heart strain and eventual heart failure. In the current study PH RNA sequencing (RNA-Seq) dataset involving 8 patients, consisting of 4 cases and 4 controls was selected for analysis. Pathway analysis revealed the involvement of the key genes in pathways crucial for PH. The protein-protein interaction (PPI) network analysis using the STRING database identified key hub genes that were significantly upregulated, including HAUS4, TUBB4A, TUBG1, NEED1, SASS6, NIN, DCUN1D3, CCDC22, ATP2B2 and LRRC37A. These hub gene-encoded proteins can be prominent drug targets for future interventions aimed at treating pulmonary hypertension.

## Background:

Pulmonary hypertension (PH) is a progressive and chronic condition that involves increased blood pressure in the pulmonary arteries, which transport deoxygenated blood from the right ventricle of the heart to the lungs to be oxygenated. PH is defined by a mean pulmonary arterial pressure (mPAP) ≥25 mmHg during rest, as determined by right heart catheterization [1]. This condition results in elevated pulmonary vascular resistance, which ultimately imposes a lot of strain on the right ventricle and, ultimately, right-sided heart failure if untreated. Pulmonary hypertension classification is into five categories based on etiology: pulmonary arterial hypertension (PAH), pulmonary hypertension secondary to left heart disease, pulmonary hypertension due to lung diseases and/or hypoxia, chronic thromboembolic pulmonary hypertension (CTEPH) and pulmonary hypertension with uncertain or multifactorial mechanisms [2]. Each of these represents unique pathophysiological mechanisms and clinical features, requiring individualized diagnostic and therapeutic strategies. For instance, vascular remodeling occurs in PAH, while left heart disease-associated PH occurs as a result of backward pressure transmission from the left atrium [3].

The burden of PH worldwide is very high as the disease impacts millions of individuals globally. The epidemiological research indicates a prevalence of 15-50 cases per million for PAH; however, the prevalence of PH in more generalized populations, such as those with heart or lung disease, is significantly higher [4, 5]. The condition is more common in women and is diagnosed frequently in elderly adults, though it can occur in any age group. The symptoms are non-specific and often related to progressive right ventricular dysfunction. The initial symptoms include shortness of breath, fatigue, weakness, angina and syncope, which are typically induced by exertion [6]. Elucidation of the molecular mechanisms of PH has highlighted key pathways participating in disease progression, including endothelial dysfunction, inflammation and smooth muscle proliferation in the pulmonary arteries [7]. These observations have fueled the progress in the field of targeted therapy, such as endothelin receptor antagonists, phosphodiesterase-5 inhibitors and prostacyclin analogs, which have proven to enhance survival and quality of life in patients with PAH. Yet most types of PH retain a dismal prognosis, particularly if they occur together with comorbidities [8]. Despite improvements in therapy, PH is still a daunting task in diagnosis and management. Limited awareness among care providers, dependency on invasive diagnostic methods and heterogeneity of disease presentation continue to hinder timely detection and treatment [9]. In addition, the inability to access specialized treatment and advanced therapies has negatively impacted the outcomes, especially in low-resource settings where the disease is often undiagnosed or mistreated. Given the variability of clinical and epidemiological manifestations of pulmonary hypertension, it is crucial to conduct research aimed at uncovering the pathophysiology, discovering biomarkers and developing new treatment approaches [10]. Therefore, it is of interest to Identification of upregulated genes in pulmonary hypertension using RNA sequencing profiling.

## Materials and Methods:

## Next-generation RNA sequencing:

Next-generation RNA sequencing samples were retrieved from the NCBI GEO Database, consisting of 8 samples: 4 treatment samples (SRR27585840, SRR27585841, SRR3929672 and SRR3929668) and 4 control samples (SRR27585842, SRR27585843, SRR3929670 and SRR3929666). The analysis was conducted using the Galaxy server. The sample datasets were imported into the server using the tool Faster Download and Extract Reads in FASTQ format from NCBI SRA (Galaxy Version 2.11.0 + galaxy0) [11]. FastQC (Galaxy Version 0.73 +galaxy0) was used to perform the read quality check and generate read quality reports. HISAT2 (Galaxy Version 2.2.1 +galaxy1), a fast and sensitive alignment tool, was used to perform sequence mapping and alignment. The human genome version hg38 was used as the reference to which the RNA sequence reads were mapped. The resulting RNA-seq data, in the form of BAM files, were analyzed using the tool Feature Counts (Galaxy version 2.0.1 + Galaxy S2) to measure the gene expression. The data was then annotated and the annotations were given in GTF file format. DESeq2 (Galaxy Version 3.50.1+galaxy0) was used to perform differential expression gene analysis based on two factors - treatment vs. control. Furthermore, ShinyGO (v0.741) was utilized to conduct in-depth Gene Ontology (GO) enrichment analysis of gene lists and retrieve visualizations of enrichment, pathways, gene characteristics and protein interactions [12].

## Protein-protein interaction network:

A protein-protein interaction (PPI) network has been constructed using STRING (12.0). STRING is a web-based database that has around 24.6 million peptides and over 3.1 billion associations, which corresponds to 5,090 different organisms [13]. To visualize the connection between the genotypes, biological systems and gene expression, Cytoscape software was used [14]. The scale-free property of the network contributed to the high scores of central nodes. Six nodes and eleven edges were found after filtering, depicting a modular structure with a Mcode score of 3.143 and a K score value of 2. Protein-protein interaction analysis was used to identify the PH hub genes and the proteins that these hub genes encode were then identified. The top hub genes were ranked using Cytohubba. HAUS4, TUBB4A, TUBG1, NEED1, SASS6, NIN, DCUN1D3, CCDC22, ATP2B2 and LRRC37A were identified as the top hub genes.

## Results:

For DESeq analysis, a total of 8 samples-four case samples and four treatment samples-were used. Treatment cases: SRR27585840, SRR27585841, SRR3929672 and SRR3929668 and controls: SRR27585842, SRR27585843, SRR3929670 and SRR3929666 are the corresponding sample IDs (Figure 1 - see PDF). Cluster analysis is then performed, which includes a heatmap depicting the sample-to-sample distance based on normalized counts. This heatmap aids in determining how similar or dissimilar the gene expression profiles of PH samples are. Dark blue in the heatmap denotes a shorter distance between samples, suggesting that the cases and controls are more alike. By highlighting functionally significant patterns in the gene expression profile, this clustering technique aids principal component regression (PCR) by identifying important components that capture the most variance and are pertinent for predicting HP outcomes (Figure 2 - see PDF). A dispersion plot was used to understand the variance of the gene expression of the gene counts (Figure 3 - see PDF). A total of 17831 differentially expressed genes were identified and among them, 9067 (down regulated genes) and 8764 (upregulated genes) were analyzed using DESeq. The significantly differentially expressed genes were identified with a threshold criterion of LogFC greater than or equal to 1.5 and a p-value less than 0.05 as the cut-off point. A total of 98 significantly upregulated DEGs were identified in PH using this threshold criterion. The PH PPI interactions between DEGs were derived from the STRING database in which the PPI network was represented in the form of nodes and edges, where each node represented a single gene's protein product and each edge represented a protein-protein connection. The network backbone of identified upregulated genes consisted of 6 nodes and 11 edges (Figure 4 - see PDF). The PPI network coefficient is a term widely used in the context of the STRING database (Search Tool for the Retrieval of Interacting Genes/Proteins) to describe the measure of the strength and reliability of interactions between proteins (PPI) inside a framework. The PPI network was visualized and identified using Cytoscape. The hub genes were found using the MCODE plugin and the parameters of DEGs clustering and scoring were as follows: for cluster 1, which consists of 6 nodes and 11 edges, MCODE score=3.143, Degree Cut-off=2, Node Score Cut-off=0.2, k-score=2 and Max. Depth=100, node density cutoff 0.1. Using cluster analysis of filtering nodes, 10 hub genes were identified. The key genes were HAUS4, TUBB4A, TUBG1, NEED1, SASS6, NIN, DCUN1D3, CCDC22, ATP2B2 and LRRC37A (Figure 5 - see PDF). The PPI network coefficient helps identify key genes involved in significant pathways. ShinyGO (v0.741), a web-based bioinformatics tool, was used to perform Gene Ontology (GO) and KEGG pathway enrichment analysis on the differentially expressed genes identified from RNA-seq data. This tool provides interactive exploration of the biological processes, molecular functions and cellular components associated with the gene list. By selecting Homo sapiens as the reference species and adjusting enrichment parameters, significant GO terms and pathways were visualized through bubble plots, network diagrams and hierarchical trees. The GO enrichment analysis revealed key biological processes significantly overrepresented in the gene list, including 10 pathways involved in microtubule polymerization, ciliary basal body-plasma membrane docking, centrosome cycle, microtubule polymerization or depolymerization, microtubule organizing center (MTOC) organization, regulation of mitotic cell cycle phase transition, microtubule cytoskeleton organization, microtubule-based process cell cycle process and, Cell cycle (Figure 6 - see PDF). Among the 10 pathways, four pathways were mainly involved in PH are microtubule polymerization, ciliary basal body-plasma membrane docking, centrosome cycle, microtubule polymerization or depolymerization. These four pathways collectively support the hypothesis that PH is involved in pulmonary vascular disease the above identified key genes and pathways may use as targets for therapeutic intervention.

## Discussion:

This study details the transcriptomic analysis of pulmonary hypertension (PH) using differential gene expression, protein-protein interaction (PPI) network modeling and pathway enrichment analysis [15]. Differential gene expression analysis was conducted across eight samples (four PH cases and four treatments) using DESeq and limma-voom. A total of 17,831 differentially expressed genes (DEGs) were identified, which included 9,067 downregulated and 8,764 upregulated genes. From amongst these, 98 significantly upregulated DEGs (LogFC ≥ 1.5, p < 0.05) were selected for further network and pathway analysis, offering insight into the molecular landscape of PH. Cluster analysis and heatmap visualization of normalized gene counts revealed distinct expression patterns between PH and control samples, validating the integrity of sample classification and underscoring the transcriptional shifts underlying the disease phenotype. This unsupervised clustering provided a foundation for subsequent principal component regression (PCR), enhancing the identification of genes contributing to the phenotypic variance. The PPI network was derived using STRING and visualized using Cytoscape. This revealed a tightly connected interaction map among significantly upregulated genes. To identify the hub genes, the backbone network, which consisted of six nodes and eleven edges, was subjected to further analysis using the MCODE plugin. Ten upregulated key genes were identified after the analysis: HAUS4, TUBB4A, TUBG1, NEED1, SASS6, NIN, DCUN1D3, CCDC22, ATP2B2 and LRRC37A (Figure 6 - see PDF). This suggests that these genes may play a central role in the pathophysiology of PH. These hub genes are notably involved in processes such as microtubule polymerization, centrosome dynamics, mitotic spindle formation and cell cycle regulation. In particular, the genes HAUS4, TUBG1, SASS6 and NIN are directly linked to spindle formation, centrosome activity and microtubule nucleation [16].

Other genes, like TUBB4A and NEK1, are linked to cell cycle regulation and cytoskeletal dynamics, which further emphasize the idea of dysregulated cell division. Genes such as DCUN1D3, CDG2, ATP2B2 and LRRC37A may also be involved in metabolic and signaling networks related to transcriptional regulation, calcium management and vascular tone [17]. Given the established link between abnormal vascular smooth muscle cell proliferations, our findings suggest that dysregulation of cytoskeletal and cell cycle machinery may drive pulmonary vascular remodeling and hypertrophy [18]. Pathway enrichment via KEGG and GO using ShinyGO (v0.741) further corroborated these observations (Figure 4 - see PDF). Significantly enriched pathways included those related to microtubule organisation, cell cycle transitions and mitotic spindle formation. These findings collectively highlighted the significance of cytoskeletal dynamics and cell division associated with many diseases [19]. This resultant enrichment is intriguing, acknowledging that the vascular remodeling in PH is repeatedly accompanied by uncontrolled cell proliferation and altered cellular architecture [20]. Remarkably, KEGG mapping also linked the upregulated DEGs to pathways related to microtubule polymerization, ciliary basal body-plasma membrane docking, centrosome cycle, microtubule polymerization or depolymerization, microtubule organizing center (MTOC) organization, regulation of mitotic cell cycle phase transition, microtubule cytoskeleton organization, microtubule-based process cell cycle process and cell cycle. These pathways are frequently linked to immune evasion, metabolic reprogramming, that are common to both PH. The intricate and multifaceted nature of PH is shown by this confluence of pathways, which may have similarities to pulmonary vascular disease. Development of targeted therapies towards these key hub genes, which play an important role in the initiation and progression of pulmonary hypertension, could help disrupt the vascular remodeling. The key genes, HAUS4, TUBB4A, TUBG1, NEK1, SASS6, NIN, DCUN1D3, CDG2, ATP2B2 and LRRC37A, may act as the therapeutic target for PH.

## Conclusion:

Ten hub genes, HAUS4, TUBB4A, TUBG1, NEK1, SASS6, NIN, DCUN1D3, CDG2, ATP2B2 and LRRC37A, were identified using DEGs analysis. These hub genes are involved in the centrosome regulation, cell cycle control and microtubule organization pathways, all of which were identified to be strongly enriched in both the GO and KEGG studies. The identified hub genes may have possibilities for additional experimental validation to develop precise and effective treatment strategies in the future.

## Disclosure statement:

No potential conflict of interest was reported by the author(s).

## Funding:

No funding was received.

## Ethical approval:

Not applicable.

## Informed consent:

Not applicable.
